# A comparative cross-sectional assessment of statistical knowledge of faculty across five health science disciplines

**DOI:** 10.1017/cts.2021.820

**Published:** 2021-07-14

**Authors:** Matthew J. Hayat, Todd A. Schwartz, MyoungJin Kim, Syeda Zahra Ali, Michael R. Jiroutek

**Affiliations:** 1 School of Public Health & Byrdine F. Lewis College of Nursing and Health Professions, Georgia State University, Atlanta, GA, USA; 2 Department of Biostatistics, Gillings School of Global Public Health & School of Nursing, University of North Carolina at Chapel Hill, Chapel Hill, NC, USA; 3 Mennonite College of Nursing, Illinois State University, Normal, IL, USA; 4 Department of Clinical Research, Department of Pharmacy Practice (adjunct appointment), College of Pharmacy & Health Sciences, Campbell University, Buies Creek, NC, USA

**Keywords:** Health sciences, biostatistics, medical education, statistics education, statistical concepts

## Abstract

**Introduction:**

The purpose of this study was to compare statistical knowledge of health science faculty across accredited schools of dentistry, medicine, nursing, pharmacy, and public health.

**Methods:**

A probability sample of schools was selected, and all faculty at each selected school were invited to participate in an online statistical knowledge assessment that covered fundamental topics including randomization, study design, statistical power, confidence intervals, multiple testing, standard error, regression outcome, and odds ratio.

**Results:**

A total of 708 faculty from 102 schools participated. The overall response rate was 6.5%. Most (94.2%) faculty reported reading the peer-reviewed health-related literature. Respondents answered 66.2% of questions correctly across all questions and disciplines. Public health had the highest performance (80.7%) and dentistry the lowest (53.3%).

**Conclusions:**

Knowledge of statistics is essential for critically evaluating evidence and understanding the health literature. These study results identify a gap in knowledge by educators tasked with training the next generation of health science professionals. Recommendations for addressing this gap are provided.

## Introduction

Healthcare researchers and practitioners rely on the peer reviewed literature to understand the state of the science and practice in a health affairs discipline. More than 95% of publications in the health literature include reporting of descriptive statistics in table or graphical form, and more than 76% of health-related publications use classical inference including statistical tests and models [1]. To understand and implement published findings, healthcare researchers and practitioners must have the knowledge, skill, and ability to understand fundamental statistical concepts and methods, as well as to critically evaluate the legitimacy and appropriateness of a study’s design, analyses, interpretations, and conclusions.

Clinicians and scientists in a health science field typically learn to read and appraise the peer reviewed literature during their graduate programs. Health science faculty are tasked with teaching them how to do this. Graduate degree program curricula are usually tightly packed with didactic, practical, and clinical education and training to address competing demands for developing substantive, science, and clinical competency. These packed graduate curricula encompass lofty goals to keep up with trends toward having more integrated, multidisciplinary content to better prepare competent professionals for an evidence-based practice and professional orientation [2–4].

Multiple studies across different health science disciplines have assessed clinicians’ statistical knowledge. We reviewed the scientific literature for studies assessing statistical knowledge in the primary health science fields of dentistry, medicine, nursing, pharmacy, and public health. For example, three studies that assessed medical resident and physician knowledge and ability to understand statistical statements in the evidence-based literature showed low levels of understanding [5–7]. In order to design graduate programs to include not only all key subject area material, but in addition the most relevant topics to create professionals able to read and understand the literature, knowing which statistical concepts and methods are used in the literature is paramount. Of note, applications of complex statistical methods from 1989 to 2005 in *New England Journal of Medicine* increased substantially [8].

Additional studies in medicine have also quantified incorrect application and usage of statistical methods [9–13]. No known assessments of statistical knowledge have been conducted in public health. Studies in the dental research literature have also documented erroneous application of statistical methods [14,15]. Several studies have also assessed statistical reporting errors in the nursing research literature [16–18]. Finally, statistical knowledge assessments of pharmacy residents and pharmacists have suggested a lack of adequate knowledge and understanding to read and comprehend the pharmacy literature [19–21].

The purpose of this study was to assess knowledge of fundamental statistical concepts among health science faculty. We focused our work on five primary health science disciplines and reviewed the types of statistical methods used in dentistry [22], medicine [23–28], nursing [18,29–30], pharmacy [31,32] and public health [1,33–35]. Based on the methods published in those fields, we based our instrument on previously published work [5,7] and focused it on key fundamental topic areas that are reported in peer reviewed journals in these respective fields. These study results provide needed information for assessing whether health science faculty are themselves adequately prepared to read and understand the literature. Since they, in turn, train health science graduate students in how to read and interpret the literature, these study findings should prove useful for identifying gaps in knowledge and training across the health science spectrum. More importantly, these data may be helpful in making informed data-based decisions about graduate health science education as well as continuing education and professional training for faculty members.

Results for the entire study sample representing responses from faculty in all five disciplines and a comparative analysis among disciplines are presented. Additional discipline-specific publications appear elsewhere for dentistry, nursing, and pharmacy [36–38].

## Methods

### Survey Development

We developed an instrument that consisted of demographic items and a statistical knowledge assessment tool with eight questions about fundamental statistical methods and concepts typically covered in a core graduate-level biostatistics course (Supplement A). The eight statistical concepts included randomization, observational studies, statistical power, confidence intervals, multiple testing, standard error, regression outcomes, and odds ratios. Each multiple-choice knowledge item had 4 choices: 1 correct answer, 2 incorrect answers, and an opt-out option to help prevent guessing. Two additional questions pertained to respondents’ attitude toward and perceived importance of statistical concepts. The data for this paper were obtained using Qualtrics (Qualtrics, Provo, UT) software to administer the instrument in an anonymous fashion [39].

The assessment questions were developed in a careful and involved process by the authors, beginning with previously published work [5,7]. The research team for this study was composed of four experienced senior biostatistics faculty members with extensive experience in teaching biostatistics. The items included in the assessment were selected through a multidimensional process. The largest focus was on statistical concepts and methods frequently reported in the health science literature. Common *biostatistics* concepts and methods typically not well understood by students that the four authors had observed through years of teaching statistics were also considered. While these topics have been thoroughly vetted through the years with our students, this is the first time we have presented these questions to faculty.

As content experts, our team of four biostatistics educators evaluated the construct validity of the items and judged the questionnaire to be assessing and measuring what we aimed to capture, namely, knowledge of fundamental statistical concepts. The content validity of the questionnaire is based on the studies we have cited from the literature of the five health science disciplines.

In addition, our process of development included consultation and input from two experienced health science faculty. Several changes were made to the questions based on their feedback, including reducing the number of responses for each knowledge question to three content-based responses. Despite our limited resources and lack of funding, we believe the research and development of the questionnaire has led to items that are straightforward and comprehensive in assessing the understanding of fundamental statistical concepts in terms of validity and reliability.

### Target Population and Study Sample

The population for this study consisted of faculty members in accredited health science schools. Table 1 displays the accrediting body and number of schools for each discipline. A total of 537 schools were targeted, with medicine representing the largest pool consisting of 147 accredited medical schools, and dentistry with the smallest pool of 66 accredited dental schools.

**Table 1.** Target population

Discipline	Accrediting Organization	Number of Schools*
Dentistry	American Dental Association	66
Medicine	Liaison Committee on Medical Education	147
Nursing	American Association of Colleges of Nursing	113
Pharmacy	Accreditation Council for Pharmacy Education	140
Public Health	Association of Schools and Programs of Public Health	71
**All Disciplines**		**537**

* Based on accredited doctoral programs listed on organization’s website in January 2017.

The study sample was obtained through probability sampling. Accredited institutions were selected using stratified random sampling with stratification by discipline. Schools within each discipline were randomly selected, and all faculty listed on each selected school’s website received an email invitation to participate. Schools were added on a rolling basis, and all faculty in each school sent two invitations. We evaluated the total target sample size every two weeks, and if this target was not achieved, additional schools were randomly selected and added, and all faculty in those schools invited. This process continued until the targeted faculty sample size for completed surveys within each discipline was reached. An *a priori* sample size calculation was based on a desired specified precision for the probability of guessing a correct answer. The probability of guessing the correct answer was based on a random guess of the three answer options for each question. We did not consider opting out of guessing in the denominator for the probability of guessing, resulting in a one-third chance of a correct guess. Applying a confidence interval half-width of 0.10, a precision estimation resulted in a minimum target sample size of 103 faculty for each discipline, or an overall minimum study sample size of 515 faculty.

### Survey Administration

A Qualtrics survey form was developed [39]. Faculty were invited to participate in an anonymous cross-sectional survey between April and August 2017. All faculty listed on the school website in each randomly selected school were sent email invitations to participate in the study. This study was reviewed and classified as exempt by the Georgia State University and the University of North Carolina at Chapel Hill Institutional Review Boards.

### Analysis

Data analyses were performed using the SAS Software System (SAS Institute, Cary, NC). Descriptive statistics were used to summarize all study measures, with mean and standard deviation for continuous variables and frequency distributions with counts and percentages for categorical variables. The main outcome in this study was the number (or percentage) of correct responses out of eight questions on the statistics knowledge assessment tool. Boxplots were constructed for number of correct responses by discipline. Statistics knowledge was scored as the total number of correct responses out of eight questions and summarized with percentages for both individual items and aggregated scores. Each question was equally weighted. Missing responses were counted as incorrect.

In addition, a general linear model was used to model the number of correct responses as a function of discipline and faculty characteristics. Model selection was done through a multitiered approach by evaluating the magnitude of each regression coefficient and assessing the contribution of each covariate to the explained variability (R^2^) of the outcome. We anticipated that faculty characteristics would differ across disciplines and so we tested for the moderating effects of discipline on the relationship between specific faculty characteristics and statistics knowledge. The use of correlation and collinearity statistics such as variance inflation factors and tolerance between predictors was used to test for multicollinearity.

## Results

Table 2 displays the sampling characteristics and response rates for the study sample. A total of 10,931 faculty in 102 schools were invited to participate. School sizes varied considerably with respect to the number of faculty. Medicine had the largest average school size with a mean of 210.2 faculty per school, and pharmacy the smallest with an average of 67.1 faculty per school. We adjusted our estimate for the number of faculty invites by subtracting the number of returned emails and delivery failure notifications from the number of invites sent out. Response rates varied across disciplines, with dentistry and medicine having the lowest response rates (4.2% and 5.5% of faculty responding, respectively). Public health had the highest response rate of 8.6%. The total study sample consisted of an overall 6.5% response rate (708 faculty respondents/10,931 adjusted number of faculty invited).

**Table 2.** Summary of sampling and response across disciplines

	Dentistry	Medicine	Nursing	Pharmacy	Public Health	All Disciplines
Number of schools sampled	16	11	26	30	19	102
Average number of faculty per sampled school	175.1	210.2	82.7	69.1	120.4	114.0
Number of faculty invited*	2,585	2,110	2,128	2,036	2,072	10,931
Number of faculty responses	109	117	164	139	179	708
**Response rate (%)**	**4.2**	**5.5**	**7.7**	**6.8**	**8.6**	**6.5**

* Adjusted for returned emails and delivery failure notifications.

### Faculty Characteristics

A summary of faculty characteristics is provided in Table 3. Females accounted for the majority of faculty respondents in nursing (90.2%) and pharmacy (64.0%), whereas males were the majority in dentistry (63.3%) and medicine (61.5%). Public health was relatively balanced (48.0% females). Prior biostatistics education was assessed with a question about the number of statistics/biostatistics courses completed. The majority of respondents in public health (75.4%) and nursing (70.7%) reported completing three or more courses. The majority of faculty among all disciplines, with the exception of public health (49.2%), reported having completed none or one epidemiology courses. Almost half of pharmacy faculty (46.8%) and medicine faculty (48.7%) reported having completed none or one biostatistics course. Thirty percent of public health respondents reported teaching statistics/biostatistics, which was about twice as many as in the other disciplines.

**Table 3.** Summary of faculty characteristics (n = 708)

Faculty characteristic	Dentistry (n = 109)	Medicine (n = 117)	Nursing (n = 164)	Pharmacy (n = 139)	Public Health (n = 179)	All Disciplines (n = 708)
	**Mean (Standard Deviation)**					
Years of professional experience	27.5 (11.6)	22.1 (13.0)	29.2 (12.7)	14.7 (10.8)	20.8 (12.4)	22.8 (13.2)
Years as a faculty member	15.7 (11.6)	16.5 (12.3)	15.0 (11.5)	10.2 (10.0)	13.6 (11.6)	13.9 (11.5)
	**Frequency (%)**					
Sex						
Male	69 (63.3)	72 (61.5)	16 (9.8)	50 (36.0)	86 (48.0)	293 (41.4)
Female	40 (36.7)	45 (38.5)	148 (90.2)	89 (64.0)	93 (52.0)	415 (58.6)
Highest Degree						
Clinical/Practice doctorate	55 (50.5)	52 (44.5)	25 (15.2)	100 (71.9)	23 (12.9)	255 (36.0)
Research doctorate	35 (32.1)	61 (52.1)	115 (70.1)	32 (23.0)	138 (77.1)	381 (53.8)
Master’s prepared	13 (11.9)	2 (1.7)	18 (11.0)	3 (2.2)	9 (5.0)	45 (6.4)
Other	6 (5.50)	2 (1.7)	6 (3.7)	4 (2.9)	9 (5.0)	27 (3.8)
Number of statistics/biostatistics courses completed						
0	16 (14.7)	17 (14.5)	0 (0)	6 (4.3)	6 (3.4)	45 (6.4)
1	32 (29.4)	40 (34.2)	13 (7.9)	59 (42.5)	13 (7.3)	157 (22.2)
2	25 (22.9)	27 (23.1)	35 (21.4)	37 (26.6)	25 (13.9)	149 (21.0)
3+	36 (33.0)	33 (28.2)	116 (70.7)	37 (26.6)	135 (75.4)	357 (50.4)
Number of epidemiology courses completed						
0	49 (45.0)	72 (61.5)	72 (43.9)	91 (65.5)	60 (33.5)	344 (48.6)
1	34 (31.2)	31 (26.5)	57 (34.8)	30 (21.6)	28 (15.7)	180 (25.4)
2	13 (11.9)	6 (5.1)	23 (14.0)	8 (5.7)	26 (14.5)	76 (10.7)
3+	13 (11.9)	8 (6.9)	12 (7.3)	10 (7.2)	65 (36.3)	108 (15.3)
Teaches statistics/biostatistics						
Yes	12 (11.0)	17 (14.5)	27 (16.5)	19 (13.7)	54 (30.2)	129 (18.2)
No	97 (89.0)	100 (85.5)	137 (83.5)	120 (86.3)	125 (69.8)	579 (81.8)
Rating of importance of statistics in role as a researcher						
Very important	67 (61.5)	83 (70.9)	124 (75.6)	89 (64.0)	148 (82.7)	511 (72.2)
Somewhat important	33 (30.3)	27 (23.1)	34 (20.7)	48 (34.5)	25 (14.0)	167 (23.6)
Not important	9 (8.2)	7 (6.0)	6 (3.7)	2 (1.5)	6 (3.3)	30 (4.2)
Reads peer-reviewed health-related scientific journal articles						
Yes	98 (89.9)	112 (95.7)	151 (92.1)	133 (95.7)	173 (96.7)	667 (94.2)
No	11 (10.1)	5 (4.3)	13 (7.9)	6 (4.3)	6 (3.3)	41 (5.8)
Attitude about fundamental statistical concepts						
Understands all expressions	53 (48.6)	79 (67.5)	119 (72.6)	92 (66.2)	157 (87.7)	500 (70.6)
Understands some expressions	46 (42.2)	34 (29.1)	42 (25.6)	42 (30.2)	21 (11.7)	185 (26.1)
Understands little/no expressions	10 (9.2)	4 (3.4)	3 (1.8)	5 (3.6)	1 (0.6)	23 (3.2)

### Importance and Attitude

In addition to the objective statistics knowledge assessment, we sought to understand how faculty rated the importance of statistics in the researcher role. Overall, 95.8% of faculty in the sample rated statistics as somewhat or very important. Most (94.2%) reported reading peer-reviewed health-related scientific journal articles. We also included a question about attitude toward understanding of fundamental statistical concepts. About half of dentistry faculty (48.6%) reported understanding all expressions of statistical concepts, whereas this result was 87.7% for public health faculty. It is also noteworthy that 9.2% of dentistry faculty reported understanding little or none of the expressions of statistical concepts listed in the attitude question, which was higher than any of the other disciplines.

### Statistics Knowledge Assessment

**Figure 1.**
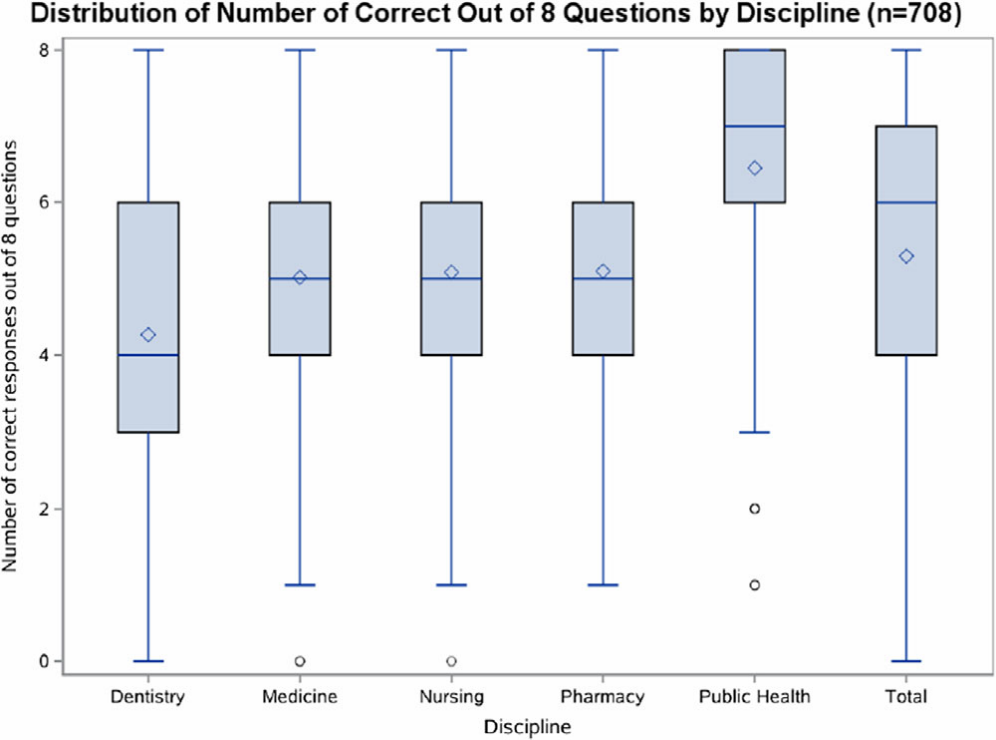
Boxplots displaying distributions for number of correct responses out of 8 questions by discipline.* *The upper and lower ends of the box are the upper and lower quartiles. The median is marked by a horizontal line inside the box. The mean is symbolized with a diamond. Circles indicate outliers.

A visual depiction of statistics knowledge assessment scores by discipline is presented (Figure 1) to display the distribution of correct responses for each discipline. Boxplots showed that the distribution for public health was shifted higher than the other disciplines and dentistry lower. The interquartile range for dentistry is substantially larger than for the other disciplines. The distributions for medicine, nursing, and pharmacy are similar to one another.

Summary statistics for statistics knowledge assessment scores by discipline are shown in Table 4. The overall percentage of correct answers across all questions and disciplines was 66.2%. For the discipline-specific results, public health performance was highest at 80.7% and dentistry lowest at 53.3%. Medicine (62.7%), nursing (63.6%), and pharmacy (63.8%) were comparable to one another. Aggregating across disciplines, the number of correct responses was highest and total scores above 80% for items dealing with observational studies, multiple testing, and standard errors. The lowest aggregated scores, which hovered around 50%, were for the items regarding randomization, confidence intervals, and odds ratios. While the overall average score for the question about understanding the difference between linear and logistic regression was 67.8%, the discipline-specific responses to this item varied considerably. Public health had an 89.9% correct response, whereas the scores for this question were much lower for dentistry (50.5%), medicine (57.3%), and pharmacy (59.0%). Interpreting an odds ratio appeared to be difficult for the majority of faculty respondents, with only public health (72.1%) scoring well above the 50% mark.

**Table 4.** Statistics knowledge assessment scores by discipline (n = 708)

	Dentistry (n = 109)	Medicine (n = 117)	Nursing (n = 164)	Pharmacy (n = 139)	Public Health (n = 179)	All Disciplines (n = 708)
	Correct Responses					
	*Frequency (%)*					
Understanding the rationale for randomization.	38 (34.9)	66 (56.4)	71 (43.3)	70 (50.4)	121 (67.6)	366 (51.7)
Describing an observational study.	80 (73.4)	90 (80.3)	135 (82.3)	111 (79.9)	164 (91.6)	584 (82.5)
Defining statistical power.	53 (48.6)	74 (63.3)	119 (72.6)	100 (71.9)	133 (74.3)	479 (67.7)
Interpreting a confidence interval.	58 (53.2)	49 (41.9)	70 (42.7)	59 (42.5)	116 (64.8)	352 (49.7)
Understanding the issue with multiple testing.	66 (60.6)	91 (77.8)	136 (82.9)	112 (80.6)	168 (93.9)	573 (80.9)
Relationship between sample size and standard error.	78 (71.6)	101 (86.3)	133 (81.1)	103 (74.1)	163 (91.1)	578 (81.6)
Understanding the difference between linear and logistic regression.	55 (50.5)	67 (57.3)	115 (70.1)	82 (59.0)	161 (89.9)	480 (67.8)
Interpreting an odds ratio.	37 (33.9)	45 (38.5)	55 (33.5)	72 (51.8)	129 (72.1)	338 (47.7)
**Overall percentage correct**	**53.3**	**62.7**	**63.6**	**63.8**	**80.7**	**66.2**

The final model is displayed in Table 5, which includes parameter estimates and 95% confidence intervals (CI). Discipline and faculty characteristics explained 40.8% of the variability of number of correct responses (R^2^ = 0.408). After controlling for faculty characteristics, dentistry had on average 1.07 fewer correct responses out of the eight statistical knowledge questions (95% CI: [−1.45, −0.68]) than that of public health. As expected, controlling for discipline and other faculty characteristics, teachers of statistics/biostatistics performed considerably better on the assessment, with instructors answering 0.91 (95% CI: [0.60, 1.21]) more items correctly, on average. Controlling for the variables in the model, faculty who reported reading the peer-reviewed health literature on average correctly answered 1.35 (95% CI: [0.86, 1.83]) more items than those that did not. Having a research doctorate was associated with statistics knowledge, with such faculty scoring on average 0.28 additional items answered correctly (95% CI: [0.02, 0.54]). However, years of professional experience appeared to have had an inverse relationship with statistics knowledge. Controlling for discipline and other faculty characteristics, for each 10 additional years of professional experience, the number of correct responses on average decreased by 0.30 additional items (95% CI: [−0.44, −0.16]). Conversely, years as a faculty member had a positive association. Controlling for other variables in the model, for each 10 additional years as a faculty member, the number of correct responses on average increased by 0.27 additional items (95% CI: [0.12, 0.42]). We tested for and failed to find a moderating effect of discipline on the relationship between each faculty characteristic and the number of correct responses.

**Table 5.** General linear model results for modeling the expected number of correct responses (out of 8 questions) as a function of discipline and faculty characteristics*

			95% Confidence Interval	
Covariate		Parameter Estimate	Lower	Upper
Discipline				
Dentistry		−1.07	−1.45	−0.68
Medicine		−0.55	−0.93	−0.18
Nursing		−0.77	−1.11	−0.42
Pharmacy		−0.40	−0.78	−0.02
Public Health		Reference		
Faculty Characteristics				
Per 10 Years of professional experience		−0.30	−0.43	−0.16
Per 10 Years as a faculty member		0.27	0.12	0.42
Research Doctorate	Yes	0.28	0.02	0.54
	No	Reference		
Biostatistics courses completed	3+	1.21	0.93	1.50
	<3	Reference		
Epidemiology courses completed	3+	0.54	0.20	0.89
	<3	Reference		
Teaches statistics/biostatistics	Yes	0.91	0.60	1.21
	No	Reference		
Reads peer-reviewed health-related scientific journal articles	Yes	1.35	0.86	1.83
	No	Reference		

* Discipline and faculty characteristics explained 40.8% of the variability of number of correct responses (R^2^ = 0.408).

## Discussion

Ours is the first study we are aware of that involved prospective data collection to assess health science faculty knowledge of fundamental statistical concepts and enable comparisons of statistics knowledge across health science disciplines. As expected, the number of biostatistics courses completed was strongly positively associated with levels of statistics knowledge, and teachers of statistics/biostatistics performed better on the knowledge assessment.

The response rate was disappointing. Despite the necessity to sample more schools than originally intended in order to obtain the planned number of faculty, we did achieve our target sample size and believe that these results accurately reflect knowledge levels across content areas and disciplines. However, there are likely some biases in our study sample. We suspect that respondents performed better than non-respondents would have as we surmise that respondents were more likely to have an interest in and regularly use statistics in their daily work than nonrespondents. Hence, it is possible that these study results overestimate levels of statistics knowledge amongst health science faculty. That 95.8% of the study sample considered statistics somewhat or very important, may reflect the respondents’ motivation to respond and participate. Although faculty were clustered within institution, institution identity data were not collected to protect anonymity and so we were unable to quantify or account for any potential clustering effect. Additionally, for the same reason, there was no way to ensure the appropriateness of academic appointment for the survey respondents. The negative effect of years of experience as a working professional on knowledge contradicted the positive effect on knowledge of years of experience as a faculty member. One possible explanation for this conflicting result is faculty who spent some of their professional years outside of academia. For example, if in a clinical setting during those years, it could explain less involvement and daily contact with research and statistical methods.

It is also noteworthy that we have likely underestimated the response rates, as our estimated response rate assumes that each email invitation was received and opened. We did not have a way of knowing whether our email invitation was received and was opened by each faculty member. Some may have been filtered into a spam folder, and others may have missed the email message without even opening it. Thus, while the denominator in our response rate calculations was based on an ideal scenario that all emails sent out were received, opened, and considered, this is very unlikely to be the case, and thus the true response rate was likely higher.

We opted for a brief eight question instrument after considering the balance of completeness of response and holding participants’ attention through completion. Each of the statistical concepts was assessed with a single question, thus limiting our understanding of faculty’s depth and scope of understanding these concepts. Our decision to limit the questionnaire to eight questions rather than a more lengthy and detailed questionnaire was made after weighing the potential benefit of more information with the risk of lower response [40–42]. In addition, we wanted to ensure that all questions included broad consideration of fundamental statistical topics relevant across these five disciplines.

We believe that the total number of correct responses out of eight questions informs and addresses our study aim regarding assessment of health science faculty knowledge of fundamental statistical concepts. It was for this reason that we applied a general linear model and fit a meaningful statistical model within this framework. The model explains 40.8% of the variability of number of correct responses, which is meaningful and informs our understanding of performance on the knowledge assessment.

There are likely a variety of factors that contribute to discipline differences in statistical knowledge and competency. We expected faculty in public health to perform better than the other disciplines, since the Association of Schools and Programs of Public Health (ASPPH) provides guidance for schools and programs with a recommended set of biostatistics competency guidelines [43]. Doctoral public health students are often required to complete at least a two-semester core biostatistics sequence, and those concentrating in epidemiology, biostatistics, or environmental science will undoubtedly complete additional biostatistics coursework. Conversely, there are no known biostatistics-specific competency guidelines in dentistry, nursing, pharmacy or medicine [44–45].

Dentistry faculty had the weakest performance on the statistics knowledge assessment, which is suggestive of the lack of biostatistics competencies in the attainment of the degree [46]. Studies of dentists have found that despite their self-perceived notions of having strong statistical knowledge, objective quantitative assessments of statistical understanding have consistently shown low levels of knowledge [7,47–48]. In fact, a study by Kim *et al.* found that more than half of the published reports in dentistry contain statistical errors [22].

The American Association of Colleges of Nursing (AACN) establishes education standards for graduate degree programs in the academic nursing discipline [49]. Unfortunately, in the very recent release of the new Essentials documents for nursing curricula, the word ‘statistics’ is not mentioned at all in the 88-page document, and statistics education or training is not mentioned in the extensive list of AACN curriculum standards. To date, no known biostatistics competency guidelines are in place for graduate nursing students [49].

The Accreditation Council for Pharmacy Education, the accrediting body for pharmacy programs in the US, published its most recent standards and key elements in 2015 [50]. Biostatistics is included among the required elements of the didactic doctor of pharmacy curriculum. However, the requirements are vague and terse: “Appropriate use of commonly employed statistical tests, management of data sets, and the evaluation of the validity of conclusions generated based on the application of those tests to the data sets.” No other mention of biostatistics appears in the standards document. However, with an increasing emphasis on research competence in doctor of pharmacy programs (Jiroutek et al, 2019 discusses this effort), most doctor of pharmacy programs now include at least one biostatistics course [38].

Our aim was to explore whether faculty had sufficient general statistics knowledge to topically read and understand the health literature. Interestingly, the vast majority of faculty surveyed reported reading the peer-reviewed health-related scientific literature. It logically follows that knowledge of fundamental statistical concepts is needed for accessibility and comprehension of what is read. Faculty respondents overwhelmingly indicated that they considered statistics important in their role as a researcher. Yet, despite the self-stated importance of statistics by the study participants, overall performance was poor, with an alarming average score of 66.2%.

It was interesting to find that years of professional experience was negatively associated with statistics knowledge. One possible explanation is that this metric serves as a proxy for the number of years since the participant was trained. If training was long ago, some or even much of that training may have been simply forgotten over time. Further, since statistical training may have increased over time in graduate health science training programs, this relationship between years of professional experience and current statistics knowledge may be muddled.

Health science faculty across all the disciplines performed poorly with public health having the highest average score and dentistry the lowest. While we cannot claim causality, we feel strongly that the specificity regarding biostatistics in the public health accreditation standards is a key factor in their faculty’s outperformance. Conversely, the lack of detail, focus, and emphasis on biostatistics education and training in accreditation standards may explain the underperformance of the others relative to public health.

Another consideration that very likely affects the statistics education process is the instructor qualification. The authors have observed that it is not uncommon for discipline-specific faculty who themselves have completed limited statistics coursework to be tapped as instructor for an introductory statistics course; yet, of course, it would be highly unusual for the converse to occur. For example, it would be highly unusual for someone without formal training in dentistry to teach dentistry. This consideration of appropriate qualifications may also be a downstream contributing factor to health science faculty’s lack of understanding observed in this study.

We expect little has changed across disciplines since 2017 when these data were collected. However, several recently published papers geared specifically toward medical research learners have the potential to affect positive change [51–53]. In the first of these papers, the authors developed a comprehensive set of statistical competencies for medical research learners [51]. The results were based on responses obtained from a prospective survey of doctoral-level statistics educators, most of whom were faculty at research institutions. Although focused on medicine, those authors provided a framework for considering statistical knowledge across the health sciences. In a follow-up study, that team assessed gaps in knowledge and training across the health spectrum [52]. Findings for their competencies labeled ‘Assess bias in publications’ and ‘Need for statistical consultation’ correspond well to our study findings, suggesting that these topics are not extensively covered in required coursework, but need to be.

## Conclusion

This work has revealed specific fundamental statistical concepts for which health science faculty exhibit substantial and common lack of understanding. Curricula would benefit from well-defined biostatistics competency guidelines and core graduate coursework in biostatistics education. Health science faculty would undoubtedly benefit from professional opportunities for enhancing their knowledge and skill in working with biostatistics concepts and methods. Training workshops and continuing education offerings should be regularly offered at institutional levels to better prepare faculty to teach and train students on evidence-based health care best practices.

## Supplementary material

For supplementary material accompanying this paper visit http://dx.doi.org/10.1017/cts.2021.820. click here to view supplementary material
