# Supplementary material for: A comparative cross-sectional assessment of statistical knowledge of faculty across five health science disciplines
Source: J Clin Transl Sci. 2021 Jul 14;5(1):e153. doi: 10.1017/cts.2021.820 (PMC8411270; doi:10.1017/cts.2021.820)
Supplement: Supplementary file 1 [file S2059866121008207sup001.docx]

**Supplement A.**

Assessment of Statistics Knowledge in the Health Sciences (**correct** **answers in bold**)

1. Which reflects your attitude about statistical concepts (e.g., standard deviation, standard error, p-values, confidence intervals, correlation coefficients)?
   1. I understand all of these expressions.
   2. I understand some of these expressions.
   3. I understand little of these expressions.
   4. I do not understand these expressions.
2. What is the rationale for random allocation in a randomized controlled trial?
3. **To produce treatment groups with similar characteristics.**
4. To ensure all subjects had an equal chance of being selected for inclusion in the study.
5. To increase the accuracy of the research results.
6. I’m not confident with my knowledge about randomization and do not want to guess.
7. Which of the following is most correct in describing an observational study?
   1. A researcher can control how subjects are assigned to groups.
   2. **The independent variable is not under the control of the researcher.**
   3. The independent variable is under the control of the researcher.
   4. I’m not confident with my knowledge about observational studies and do not want to guess.
8. Which of the following best describes statistical power?
   1. Ineffective treatment is found to be ineffective.
   2. Effective treatment is declared ineffective.
   3. **Effective treatment is declared effective.**
   4. I am not familiar with statistical power and do not want to guess.
9. How would you interpret a 95% confidence interval for the true mean of a numeric health outcome?
   1. You can be 5% confident that the interval will not include the true mean.
   2. You can be 95% confident that the interval will include the true mean.
   3. **If you draw repeated random samples and calculate a confidence interval for each, you can expect 95% of the intervals to contain the true mean.**
   4. I do not understand the expression and do not want to guess.
10. Which of the following is correct if one of 15 t-tests is significant at the .05 level?
    1. This is a result worthy of publication.
    2. **You should exercise caution in interpreting this single significant finding, since the Type I error rate is likely inflated due to multiple testing.**
    3. You should consider a different statistical analysis since you only have one statistically significant result.
    4. I do not understand the expression and do not want to guess.
11. What is your understanding of how the standard error will change as the sample size increases?
    1. **Standard error will decrease.**
    2. Standard error will remain the same.
    3. Standard error will increase.
    4. I do not understand the expression and do not want to guess.
12. Which of the following best describes the primary difference between linear regression and logistic regression?
    1. The dependent variable is categorical in a linear regression while it is continuous in logistic regression.
    2. **The dependent variable is continuous in a linear regression while it is categorical in logistic regression.**
    3. They serve the same purpose so can be used interchangeably.
    4. I do not understand regression and do not want to guess.
13. What is the most correct interpretation of an odds ratio of 3.0?
    1. There is a statistically significant association (at the 0.05 level) between the exposure and the outcome.
    2. The likelihood of the event is 3 times higher in one group than another.
    3. **The odds of the event is 3 times as high in one group as in the other.**
    4. I do not understand odds ratios and do not want to guess.
14. How important is understanding of statistical concepts for you in your role as a researcher?
15. Very important
16. Somewhat important
17. Not important
